# Effect of oocyte chromatin status in porcine follicles on the embryo development *in vitro*

**DOI:** 10.5713/ajas.18.0739

**Published:** 2019-01-02

**Authors:** Joo Bin Lee, Min Gu Lee, Tao Lin, Hyeon Yeong Shin, Jae Eun Lee, Jung Won Kang, Dong-Il Jin

**Affiliations:** 1 Division of Animal and Dairy Science, Chungnam National University, Daejeon 34134, Korea

**Keywords:** Porcine Oocyte Chromatin, Follicle Size, Oocyte Maturation, Embryo Development

## Abstract

**Objective:**

The main goal of this study was to provide a morphological indicator that could be used to select high-quality oocytes of appropriate meiotic and developmental capabilities in pig. The higher quality of immature oocytes, the higher success rates of *in vitro* maturation (IVM) and *in vitro* fertilization (IVF). Thus, prior to the IVM culture, it is important to characterize oocytes morphologically and biochemically in order to assess their quality. Two of the largest indicators of oocyte quality are the presence of cumulus cells and status of chromatin. To investigate the effects of porcine oocyte chromatin configurations on the developmental capacity of blastocysts, we assessed oocyte chromatin status according to follicle size and measured the developmental potency of blastocysts.

**Methods:**

To sort by follicle size, we divided the oocytes into three groups (less than 1 mm, 1 to 3 mm, and more than 3 mm in diameter). To assess chromatin configuration, the oocytes were assessed for their stages (surrounded nucleolus [SN] germinal vesicle [GV], non-surrounded nucleolus [NSN] GV, GV breakdown, metaphase I [MI], pro-metaphase II [proMII], and metaphase II [MII]) at different maturation times (22, 44, and 66 h). To assess the development rate, oocytes of each follicle size were subjected to parthenogenetic activation for further development. Finally, GV oocytes were grouped by their chromatin configuration (SN, SN/NSN, and NSN) and their global transcriptional levels were measured.

**Results:**

SN GV oocytes were more suitable for IVF than NSN GV oocytes. Moreover, oocytes collected from the larger follicles had a greater distribution of SN GV oocytes and a higher developmental capacity during IVM, reaching MII more quickly and developing more often to blastocysts.

**Conclusion:**

Porcine oocytes with high-level meiotic and developmental capacity were identified by analyzing the relationship between follicle size and chromatin configuration. The porcine oocytes from large follicles had a significantly higher SN status in which the transcription level was low and could be better in the degree of meiotic progression and developmental capacity.

## INTRODUCTION

In most mammals, pre-ovulatory oocytes stall at the diplotene stage of meiosis prophase I until they proceed to ovulation or atresia [1,2]. The resumption of meiosis in ovulatory oocytes can be triggered *in vitro* under appropriate culture conditions; to date, *in vitro* fertilization (IVF) embryos have been produced from *in vitro* maturation (IVM) oocytes through these procedures. Efficient production of embryos from IVM oocytes requires the use of high-quality oocytes, and researchers have sought to optimize the conditions for their *in vitro* production. As one would expect, the quality of immature oocytes is an important determinant for the quality of matured oocytes. Thus, morphological and biochemical analyses have been used to distinguish high-quality immature oocytes [3,4]. Previous studies have reported that the follicle sizes and chromatin configurations of mammalian oocytes are related to their ability to complete meiosis [5].

There are species-specific differences in the developmen tal capacity of mature oocytes during antral folliculogenesis. Moreover, researchers have identified different timings for folliculogenesis in the mouse [6–8], pig [9,10] and cow [11,12]. Pre-ovulatory oocytes whose nuclei are stalled at prophase I of meiosis are called germinal vesicles (GVs). In antral follicles, GV oocytes may be separated two types based on their chromatin configuration [3,4,13,14]: the surrounded nucleolus (SN) type, in which the GV chromatin forms a ring around the nucleolus; and the non-surrounded nucleolus (NSN) type, in which the GV chromatin is diffused around the nucleolus. The nuclei of most oocytes near ovulation are found to be in the SN chromatin configuration, whereas those of oocytes in the pre-antral follicle stage tend to be in the NSN chromatin configuration [3,13]. These features have been observed for the pre-ovulatory oocytes of various species, including mouse [7,15], rat [16], pig [17], monkey [18], and human [19,20]. In mice, many GV oocytes from antral follicles cease their total transcriptional activity 2 h before GV breakdown (GVBD) begins. This reflects their chromatin status, as NSN oocytes retain their transcriptional activity, whereas SN oocytes exhibit inactivation of RNA polymerase II-dependent RNA synthesis [14,21]. Consistent with these observations, porcine oocytes reportedly exhibit decreased transcriptional activity as their development progresses [22].

To elucidate the effects of porcine oocyte chromatin con figuration on the developmental capacity, we performed morphological classification of immature porcine oocytes and examined whether their meiosis and/or development differed with respect to the studied parameters (size and the chromatin configuration). We also analyzed the relationship of the GV chromatin configuration and the generation of embryos from oocytes subjected to IVM and parthenogenetic activation.

## MATERIALS AND METHODS

### Chemicals

All chemicals used in this study were purchased from Sigma (Sigma-Aldrich, St. Louis, MI, USA) unless otherwise noted.

### *In vitro* maturation of porcine oocytes

All animal experiments were approved by the Institutional Animal Care and Use Committee of Chungnam National University. Pig ovaries were collected at a local slaughterhouse from gilts weighing between 100 and 130 kg. Within 3 h, the ovaries were and transported to the laboratory at 37°C in 0.9% (w/v) saline supplemented with 75 μg/mL potassium penicillin G and 50 mg/mL streptomycin sulfate. The ovaries were washed in fresh saline and cumulus oocyte complexes (COCs) were separated from follicles of three different sizes (diameters): less than 1 mm, 1 to 3 mm, and 3 to 6 mm. Follicles smaller than 1 mm were aspirated using a 21-gauge needle, while the others were aspirated using an 18-gauge needle. After being washed three times in TL-HEPES medium [23], oocytes of three groups were allowed to mature in IVM medium in four-well multi-dishes (500 μL/well) (Nunc, Roskilde, Denmark) at 38.5°C under 5% (v/v) CO_2_ in air. The IVM medium consisted of North Carolina State University Medium-23 [24] supplemented with 10% (v/v) follicular fluid, 0.57 mM cysteine, 10 ng/mL β-mercaptoethanol, 10 ng/mL epidermal growth factor, 10 IU/mL pregnant mare serum gonadotropin, and 10 IU/mL human chorionic gonadotropin. The oocytes were cultured for 22 h, washed three times with IVM medium, and cultured for another 22 h in hormone-free IVM medium.

### Morphological evaluation of germinal vesicle oocytes

The immature oocytes of the three groups were vortexed and pipetted in 0.1% hyaluronidase solution to denude them of cumulus cells. No vortex step was required for morphological evaluation of oocytes subjected to IVM for 22 or 44 h. The prepared oocytes were stained with 4′,6-diamidino-2-phenylindole (DAPI). Images were obtained using a scanning laser confocal microscope (Zeiss, Jena, Germany) and analyzed employing the LSM Image Browser software (Zeiss, Jena, Germany).

### Parthenogenetic activation

After IVM, cumulus cells were removed by repeated pipetting in TL-HEPES supplemented with 0.1% (w/v) polyvinyl alcohol (PVA) and 0.3% (w/v) hyaluronidase. The oocytes were transferred to activation solution [0.3 M D-mannitol, 0.1 mM MgSO_4_, 0.05 mM CaCl_2_, and 0.01% (w/v) PVA] and stimulated with a direct current pulse of 15 kV/cm for 30 us, which was applied using a BTX Elector-Cell Manipulator 2001 (BTX, San Diego, CA, USA). After activation, oocytes were washed, transferred into 50 μL of PZM-3 medium, covered with mineral oil in a 60-mm dish (Thermo Fisher Scientific, Waltham, MA, USA), and cultured at 38.5°C under 5% (v/v) CO_2_ for 7 days.

### Fix and immunostaining

#### Fix and DAPI staining

GV, GVBD, metaphase I (MI), and metaphase I (MII) oocytes were collected at 0, 22, and 44 h of IVM culture. Blastocyst-stage embryos were obtained on day 7 after the activation of IVM oocytes. The oocytes and embryos were fixed in 4% (v/v) paraformaldehyde for 30 min at room temperature and permeabilized with 0.1% (v/v) Triton-100 for 30 min. After three washes, the various samples were mounted using vectashield mounting medium containing DAPI (Vector laboratories, Burlingame, CA, USA). Images were analyzed using a Zeiss scanning laser confocal microscope and the LSM Image Browser software (Zeiss, Germany).

#### Immunostaining and in situ run-on transcription

For transcription labeling, oocytes were cultured in IVM medium plus 5 mM 5-fluorouridine for 1 h [25]. Then NSN, SN/NSN, and SN oocytes were collected and fixed. Fix method was described in fix and DAPI staining. Fixed oocytes were blocked non-specific binding sites with 3% (w/v) bovine serum albumin (BSA) for 20 minutes, followed by 5 minutes in PBG [PBS containing 0.5% (w/v) BSA and 0.1% (w/v) gelatin from the skin of cold-water fish]. Next, oocytes were subsequently washed three times, for 5 min each time in PBG. And incubated with the fluorescein isothiocyanate-conjugated goat anti-mouse immunoglobulin G secondary antibody (Santa Cruz Biotechnologies, Dallas, TX, USA) for 1 h in PBG at room temperature. Next, oocytes were washed gradually three times in PBG and PBS for 3 minutes each time. To observe the stained oocytes using microscopy, stained oocytes were put on slides and mounted under coverslips using Vectashield mounting medium containing DAPI.

### Statistical analysis

All experiments were replicated more than three times. All data were analyzed using analysis of variance in the SPSS software package (version 20; IBM, Armonk, NY, USA). Duncan’s multiple range test was used to test for significance between groups according to follicle size, and a p-value <0.05 was considered to be statistically significant.

## RESULTS

### Relationship between follicle size and the chromatin configuration of harvested germinal vesicle oocytes

**Figure 1:**
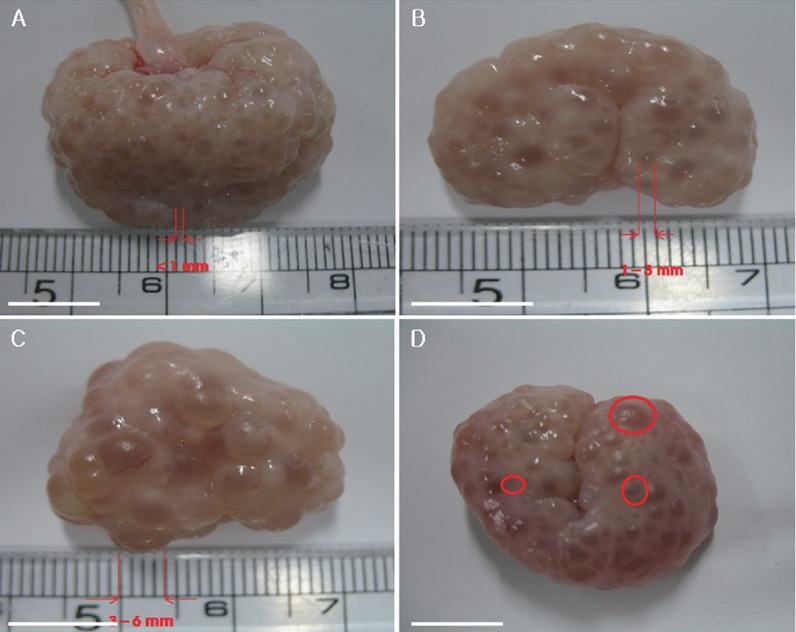
Immature porcine oocytes were classified into three groups based on the sizes of the follicles from which they were collected. Oocytes collected from follicles of less than 1 mm (A), 1 to 3 mm (B), 3 to 6 mm (C) and image of follicles of all three sizes in a single ovary (D). Scale bar = 1 cm.

**Figure 2:**
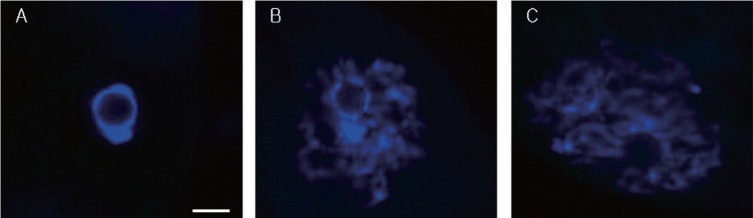
Representative images of the three possible chromatin configurations in porcine oocytes. Oocytes were staining with DAPI. Shown are the SN configuration (A), the intermediate configuration of SN and NSN (SN/NSN) (B), and the NSN configuration (C). DAPI, 4′,6-diamidino-2-phenylindole; SN, surrounded nucleolus; NSN, non-surrounded nucleolus. Scale bar = 10 μm.

To study the relationship between GV chromatin configuration and follicle size, immature oocytes were divided into three groups based on having been collected from small (less than 1 mm), medium (1 to 3 mm) and large (3 to 6 mm) follicles (Figure 1), and their chromatin configurations (SN, SN/NSN, or NSN) were observed using DAPI staining and fluorescence microscopy (Figure 2). The oocytes of the small follicle groups were predominantly NSN (64.3%±4.0%), with smaller proportions of SN (28.0%±1.9%) and SN/NSN (7.7%±2.2%) (Table 1). Those of the medium follicle group were NSN (49.4%± 1.6%) followed closely by SN (40.6%±5.6%), with a smaller proportion of SN/NSN (10.1%±4.3%). Finally, the oocytes of the largest follicle group were predominantly SN (65.2%±5.5%), followed by NSN (29.8%±6.1%), and SN/NSN (5.1%±2.9%). The large follicle group had a significantly higher SN ratio and a significantly lower NSN ratio than the small and medium follicle groups. There was no significant difference in the SN or NSN ratios of the small and medium follicle groups, or in the SN/NSN ratio across all three groups.

### Meiotic progress of immature oocytes after 22 h of *in vitro* maturation

**Figure 3:**
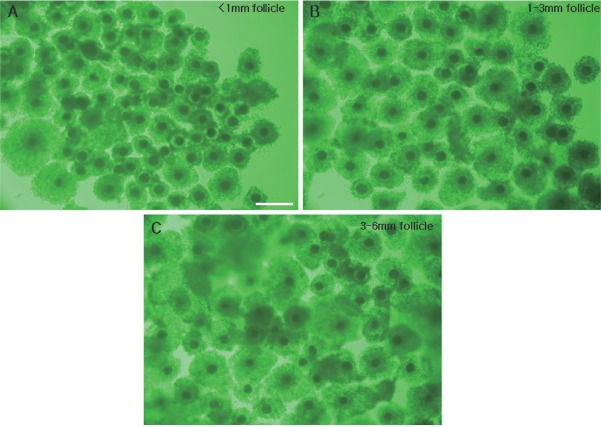
Comparison of COCs of 22 h IVM porcine oocytes derived from follicles of the three different sizes. COCs from follicles of less than 1 mm (A), 1 to 3 mm (B), and 3 to 6 mm (C). COCs, cumulus-oocyte complexes; IVM, *in vitro* maturation. Scale bar = 500 μm.

**Figure 4:**
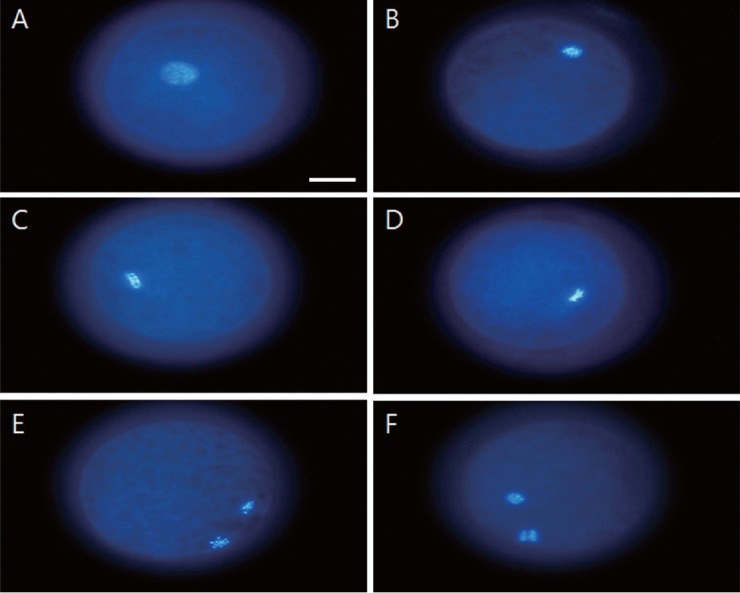
Chromatin configurations of porcine oocytes in different meiotic stages. Chromatin were counterstained with DAPI. Chromatin configuration of germinal vesicle breakdown (A). Chromatin configuration during metaphase I (B). Chromatin configuration during pro-metaphase II (C, D). Chromatin configuration during metaphase II (E, F). DAPI, 4′,6-diamidino-2-phenylindole. Scale bar = 30 μm.

We observed the distribution of oocytes in the various stages of meiotic meiosis according to follicle size after 22 h of incubation (Table 2). Oocytes of the small, medium and large follicle groups were observed under a microscope (Figure 3), and the chromatin was identified as corresponding to GV, GVBD, MI, proMII, or MII (see Figure 4 for representative images). At this time point, MI oocytes predominated in the small, medium and large follicle groups (68.4%±1.2%, 64.1% ±0.6%, and 53.7%±1.7%, respectively). And proMII oocytes predominated in the large, medium and small follicle groups (39.0%±1.9%, 27.3%±0.4%, and 12.2%±1.1%) Also, MII oocytes predominated in the large, medium and small follicle groups (7.4±1.0%, 3.8%±0.4%, and 0%). There appeared to be a positive relationship between the follicle size and degree of meiotic progression. For example, the small follicle group was the only one to retain GV-stage oocytes.

### Meiotic progress of immature oocytes after 44 h of *in vitro* maturation

**Figure 5:**
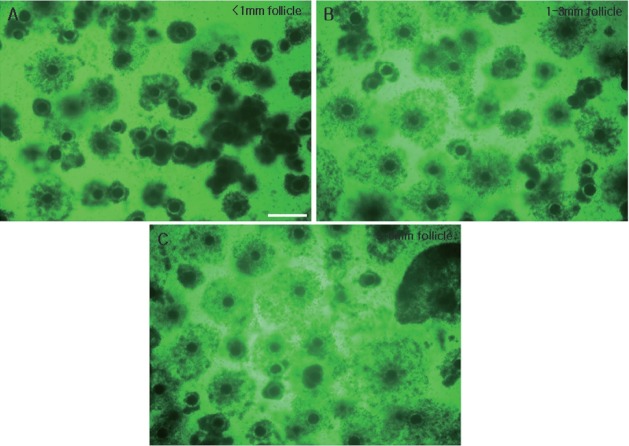
Comparison of COCs of 44 h IVM porcine oocytes derived from follicles of the three different sizes. COCs from follicles of less than 1 mm (A), 1 to 3 mm (B), and 3 to 6 mm (C). COCs, cumulus-oocyte complexes; IVM, *in vitro* maturation. Scale bar = 500 μm.

We observed the distribution of oocytes in the various stages of meiotic meiosis according to follicle size after 44 h of incubation (Table 3) and observed the oocytes under a microscope (Figure 5). In the small follicle group, the most common stage was MI (54.3%±2.0%), medium follicle group was MII (46.4% ±0.5%) and large follicle group was MII (80.7%±1.9%). We could observe that the greater the size of the follicles, the more progression of meiosis. In addition, we can observe that the meiosis progressed more than 22 h when the ratio of MII increased greatly. In addition, we can observe that all oocytes of GV stage disappear from the small group.

### Meiotic progress of immature oocyte after 66 h *in vitro* maturation

**Figure 6:**
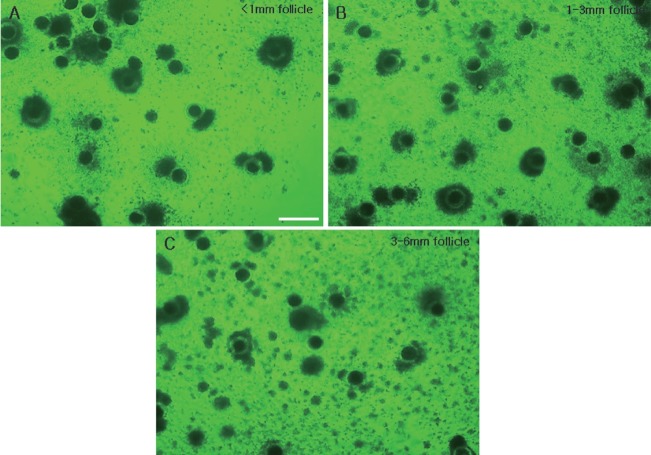
Comparison of COCs of 66 h IVM porcine oocytes derived from follicles of the three different sizes. COCs from follicles of less than 1 mm (A), 1 to 3 mm (B), and 3 to 6 mm (C). COCs, cumulus-oocyte complexes; IVM, *in vitro* maturation. Scale bar = 500 μm.

We observed the distribution of meiotic meiosis according to follicle size after 44 h incubation (Table 4). In addition, groups according to the size of each follicle were observed under a microscope (Figure 6). Most frequent stage in small follicle group was MII stage (65.2%±1.0%), medium follicle group was MII (81.2%±0.8%) and large follicle group was MII (97.2% ±0.6%). We could observe that the greater size of follicles, the more progression of meiosis. In addition, we can observe that the meiosis progressed more than 44 h when the ratio of MII increased greatly. In the groups other than small group, we could see that oocytes of the GVBD stage disappeared.

### Development of parthenogenetic embryos derived from different follicular sizes

Developmental rates of parthenogenetic embryos derived from different follicular sizes (Table 5). The blastocyst rate of oocytes can be seen to be significantly lower in the remaining groups when compared to the larger group. The medium group was also higher than the small group. In addition, the rates of cleavage, morula, and blastocyst were significantly higher in the large group compared to the other groups.

### Transcriptional status in germinal vesicle oocytes

**Figure 7:**
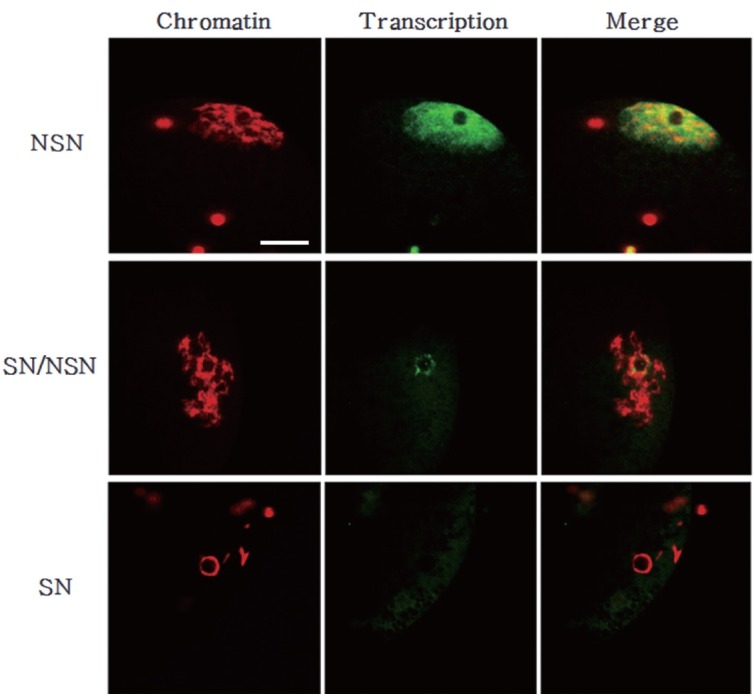
Chromatin configuration and total transcription level in growing and fully-grown oocytes. Chromatin was counterstained with DAPI (red). Transcription was examined by monitoring the incorporation of 5-fluorouridin into nascent RNAs (green). Note that transcription is lower in SN/NSN and SN oocytes than in NSN oocytes. DAPI, 4′,6-diamidino-2-phenylindole; SN, surrounded nucleolus; NSN, non-surrounded nucleolus. Scale bar = 30 μm.

Finally, we analyzed the total transcriptional activity of GV oocytes according their chromatin configurations (Figure 7). Interestingly, oocytes with the NSN chromatin configuration exhibited vigorous transcriptional activity. And that it even came out of the nucleus. Conversely, the transcription level was low in oocytes with the SN chromatin configuration. The transcription level of oocytes with NSN/SN chromatin was intermediate between those of the other two groups.

## DISCUSSION

The quality and developmental capacity of immature oocytes is very important for experiments involving the *in vitro* production of embryos. Researchers have studied the developmental capacity of immature oocytes in relation to various factors, but it remains difficult to predict the developmental potential of immature oocytes. The use of external factors to identify damaged oocytes could help improve the efficiency of reproductive technology. Here, we set out to evaluate the oocyte morphological indicators for high-quality oocytes of appropriate meiotic and developmental capabilities in pig. Toward this end, we compared the chromatin configuration of GV oocytes with the sizes of the follicles from which they were obtained, examined the abilities of oocytes from the different follicle size groups to progress through meiosis during IVM, and analyzed the development rates for parthenogenetic activation-derived embryos arising from oocytes of the different follicle size groups. Finally, we evaluated the total transcription levels of GV oocytes in each of the follicle size groups.

The developmental capacity of immature oocytes, which reflects the size of the original follicle and the shape and quality of oocytes during their early *in vitro* processing, is associated with the global efficiency of *in vitro* embryo production. This topic has been extensively studied in various animal species [26–29]. One way to assess the quality of immature oocytes is to identify their chromatin configuration. Motlik and Fulka [30] first observed the chromatin configuration of GV oocytes in porcine the authors identified four chromatin configurations of GV oocytes (GV1 to 4) based on the status of the chromatin, nucleolus and orcein-stained nuclear membrane. Since then, the chromatin configurations of porcine GV oocytes have been reported during folliculogenesis [31,32].

In this study, we observed the chromatin configuration of porcine GV oocytes using DAPI staining. The GV oocytes were divided into three groups according to the sizes of their original follicles (less than 1 mm, 1 to 3 mm, 3 to 6 mm in diameter) and classified by three chromatin patterns (SN, SN/NSN, and NSN) observed upon DAPI staining. The NSN chromatin composition was significantly more frequent than the other configurations in the small follicle group, whereas this composition was significantly less frequent than the other configurations in the large follicle group. The SN chromatin composition was significantly more frequent in the large follicle group than in the small and medium groups. There was no significant difference in the frequency of the SN or NSN configurations in the small and medium follicle groups. As follicle size and oocyte diameter are important factors in determining the maturation and developmental capacity of oocytes [28], our results show that the porcine oocytes from large follicles had a significantly higher SN status in which the transcription level was low compared with those with NSN status. Growing oocytes transcribe RNAs that encode necessary information for the early stages of embryonic development; these are then stored or translated into proteins. Transcription activity is terminated upon GVBD, and oocytes undergo the series of chromosomal condensation steps that lead to maturation [32]. Here, we analyzed the transcription levels of porcine GV oocytes derived from the different follicle size groups and found that the NSN oocytes exhibited an even distribution of transcription throughout the nucleus, whereas SN oocytes exhibited much lower levels of transcription.

We also monitored the progression of meiosis during the growth and IVM (0, 22, 44, and 66 h) of oocytes of the three groups, as well as their ability to develop to embryos following parthenogenetic activation. The blastocyst rate was higher in the large follicle group compared to the small and medium groups, and that of the medium follicle group was higher than that of the small follicle group. Overall, the embryo developmental capacity was highest among oocytes of the large follicle group.

In sum, our present study shows that we can identify oo cytes with high-level meiotic and developmental capacity by analyzing the relationship between follicle size and chromatin configuration. The porcine oocytes from large follicles had a significantly higher SN status in which the transcription level was low, could be better in the degree of meiotic progression and developmental capacity.

## Figures and Tables

**Table 1:** Chromatin configuration composition of porcine oocytes derived from follicles with different diameters

Diameter of follicle (mm)	No. of examined oocytes	No. of oocytes (%±SE)		

		NSN	SN/NSN	SN
Small (<1 mm)	350	225 (64.3±4.03^a^)	27 (7.7±2.17)	98 (28.0±1.86^a^)
Medium (1–3 mm)	397	196 (49.4±1.61^a^)	40 (10.1±4.30)	161 (40.6±5.60^a^)
Large (3–6 mm)	396	118 (29.8±6.14^b^)	20 (5.1±2.93)	258 (65.2±5.54^b^)

Experiments were repeated three times.

SE, standard error; NSN, non-surrounded nucleolus; SN, surrounded nucleolus.

a,b Values with different superscripts in the same column are significantly different (p<0.05).

**Table 2:** Meiotic progress of oocytes derived from follicles of different diameters after 22 h culture *in vitro*

Diameter of follicle (mm)	No. of examined oocytes	No. of oocytes (%±SE)					

		GV		GVBD	MI	Pro MII	MII

		NSN	SN				
Small (<1 mm)	278	0 (0)	13 (4.68±0.88^a^)	41 (14.75±1.75^a^)	190 (68.35±1.22^a^)	34 (12.23±1.13^a^)	0 (0^a^)
Medium (1–3 mm)	315	0 (0^b^)		15 (4.76±0.86^b^)	202 (64.13±0.58^a^)	86 (27.30±0.37^b^)	12 (3.81±0.35^b^)
Large (3–6 mm)	244	0 (0^b^)		0 (0^c^)	131 (53.69±1.67^b^)	95 (38.93±1.88^c^)	18 (7.38±0.98^c^)

Experiments were repeated three times.

SE, standard error; GV, germinal vesicle; NSN, non-surrounded nucleolus; SN, surrounded nucleolus; GVBD, germinal vesicle breakdown; MI, metaphase I; Pro MII, pro-metaphase II; MII, metaphase II.

a–c Values with different superscripts in the same column are significantly different (p<0.05).

**Table 3:** Meiotic progress of oocytes derived from follicles of different diameters after 44 h culture *in vitro*

Diameter of follicle (mm)	No. of examined oocytes	No. of embryos (%±SE)				

		GV	GVBD	MI	Pro MII	MII
Small (<1 mm)	245	0	16 (6.53±0.46^a^)	133 (54.29±2.02^a^)	42 (17.14±1.21^a^)	54 (22.04±1.50^a^)
Medium (1–3 mm)	194	0	1 (0.52±0.68^b^)	72 (37.11±1.42^b^)	31 (15.98±0.41^a^)	90 (46.39±0.52^b^)
Large (3–6 mm)	218	0	0 (0^b^)	29 (13.30±0.42^c^)	13 (5.96±0.81^b^)	176 (80.73±1.89^c^)

Experiments were repeated three times.

SE, standard error; GV, germinal vesicle; GVBD, germinal vesicle breakdown; MI, metaphase I; Pro MII, pro-metaphase II; MII, metaphase II.

a–c Values with different superscripts in the same column are significantly different (p<0.05).

**Table 4:** Meiotic progress of oocytes derived from follicles of different diameters after 66 h culture *in vitro*

Diameter of follicle (mm)	No. of examined oocytes	No. of embryos (%±SE)				

		GV	GVBD	MI	Pro MII	MII
Small (<1 mm)	210	0	3 (1.43±0.87)	46 (21.90±3.44^a^)	24 (11.43±2.99^a^)	137 (65.24±1.02^a^)
Medium (1–3 mm)	213	0	0	27 (12.68±1.04^b^)	13 (6.10±0.34^ab^)	114 (81.22±0.79^b^)
Large (3–6 mm)	176	0	0	3 (1.70±0.88^c^)	2 (1.14±0.59^b^)	123 (97.16±0.61^c^)

Experiments were repeated three times.

SE, standard error; GV, germinal vesicle; GVBD, germinal vesicle breakdown; MI, metaphase I; Pro MII, pro-metaphase II; MII, metaphase II.

a–c Values with different superscripts in the same column are significantly different (p<0.05).

**Table 5:** Developmental competence of parthenogenetic embryos derived from porcine follicles of different sizes

Diameter of follicle (mm)	No. of examined oocytes	No. of embryo (%±SE)		

		Cleaved	Morula	Blastocysts
Small (<1 mm)	502	422 (84.06±1.89^a^)	202 (40.24±1.56^a^)	51 (10.16±0.25^a^)
Medium (1–3 mm)	408	363 (88.97±1.80^ab^)	203 (49.75±1.57^b^)	76 (18.63±1.19^b^)
Large (3–6 mm)	383	352 (91.91±1.80^b^)	215 (56.14±1.06^c^)	104 (27.15±0.60^c^)

Experiments were repeated four times.

SE, standard error.

a–c Values with different superscripts in the same column are significantly different (p<0.05).

## References

[1] Pincus G, Enzmann EV (1935). The comparative behavior of mammalian eggs *in vivo* and *in vitro*. I. The activation of ovarian eggs. J Exp Med.

[2] Edwards RG (1965). Maturation *in vitro* of mouse, sheep, cow, pig, rhesus monkey and human ovarian oocytes. Nature.

[3] Debey P, Szollosi MS, Szollosi D, Vautier D, Girousse A, Besombes D (1993). Competent mouse oocytes isolated from antral follicles exhibit different chromatin organization and follow different maturation dynamics. Mol Reprod Dev.

[4] Zuccotti M, Piccinelli A, Giorgi Rossi P, Garagna S, Redi CA (1995). Chromatin organization during mouse oocyte growth. Mol Reprod Dev.

[5] Tan JH, Wang HL, Sun XS, Liu Y, Sui HS, Zhang J (2009). Chromatin configurations in the germinal vesicle of mammalian oocytes. Mol Hum Reprod.

[6] Eppig JJ, Schroeder AC (1989). Capacity of mouse oocytes from preantral follicles to undergo embryogenesis and development to live young after growth, maturation and fertilization *in vitro*. Biol Reprod.

[7] Zuccotti M, Ponce RH, Boiani M (2002). The analysis of chromatin organisation allows selection of mouse antral oocytes competent for development to blastocyst. Zygote.

[8] Pesty A, Broca O, Poirot C, Lefèvre B (2008). The role of PLC beta 1 in the control of oocyte meiosis during folliculogenesis. Reprod Sci.

[9] Motlik J, Fulka J, Fléchon JE (1986). Changes in intercellular coupling between pig oocytes and cumulus cells during maturation *in vivo* and *in vitro*. J Reprod Fertil.

[10] Wu D, Cheung QC, Wen L, Li J (2006). A growth-maturation system that enhances the meiotic and developmental competence of porcine oocytes isolated from small follicles. Biol Reprod.

[11] Pavlok A, Lucas-Hahn A, Niemann H (1992). Fertilization and developmental competence of bovine oocytes derived from different categories of antral follicles. Mol Reprod Dev.

[12] Lodde V, Modina S, Galbusera C, Franciosi F, Luciano AM (2007). Large-scale chromatin remodeling in germinal vesicle bovine oocytes: interplay with gap junction functionality and developmental competence. Mol Reprod Dev.

[13] Mattson BA, Albertini DF (1990). Oogenesis: chromatin and microtubule dynamics during meiotic prophase. Mol Reprod Dev.

[14] Bouniol-Baly C, Hamraoui L, Guibert J, Beaujean N, Szöllösi MS, Debey P (1999). Differential transcriptional activity associated with chromatin configuration in fully-grown mouse germinal vesicle oocytes. Biol Reprod.

[15] Wickramasinghe D, Ebert KM, Albertini DF (1991). Meiotic competence acquisition is associated with the appearance of M-phase characteristics in growing mouse oocytes. Dev Biol.

[16] Mandl AM (1963). Pre-ovulatory changes in the oocyte of the adult rat. Proc R Soc Lond B Biol Sci.

[17] Crozet N, Motlik J, Szollosi D (1981). Nucleolar fine structure and RNA synthesis in porcine oocytes during the early stages of antrum formation. Biol Cell.

[18] Lefèvre B, Gougeon A, Nomé F, Testart J (1989). *In vivo* changes in oocyte germinal vesicle related to follicular quality and size at mid-follicular phase during stimulated cycles in the Cynomolgus monkey. Reprod Nutr Dev.

[19] Tesarik J, Travnic P, Kopecny V, Kristek F (1983). Nucleolar transformations in the human oocyte after completion of growth. Gamete Res.

[20] Parfenov V, Potchukalina G, Dudina L, Kostyuchek D, Gruzova M (1989). Human antral follicles: oocyte nucleus and the karyosphere formation (electron microscopic and autoradiographic data). Gamete Res.

[21] De La Fuente R, Eppig JJ (2001). Transcriptional activity of the mouse oocyte genome: companion granulosa cells modulate transcription and chromatin remodeling. Dev Biol.

[22] Bjerregaard B, Wrenzycki C, Philimonenko VV (2004). Regulation of ribosomal RNA synthesis during the final phases of porcine oocyte growth. Biol Reprod.

[23] Funahashi H, Cantley T, Day BN (1994). Different hormonal requirements of pig oocyte-cumulus complexes during maturation *in vitro*. J Reprod Fertil.

[24] Petters RM, Wells KD (1993). Culture of pig embryos. J Reprod Fertil Suppl.

[25] Oqani RK, Lee MG, Diao YF, Xun R, Jin DI (2013). Halogenated nucleotide labeling of nascent RNAs reveals dynamic transcription in growing pig oocytes. Dev Dyn.

[26] Hirao Y, Nagai T, Kubo M, Miyano T, Miyake M, Kato S (1994). *In vitro* growth and maturation of pig oocytes. J Reprod Fertil.

[27] Crozet N, Ahmed-Ali M, Dubos MP (1995). Developmental competence of goat oocytes from follicles of different size categories following maturation, fertilization and culture *in vitro*. J Reprod Fertil.

[28] Marchal R, Vigneron C, Perreau C, Bali-Papp A, Mermillod P (2002). Effect of follicular size on meiotic and developmental competence of porcine oocytes. Theriogenology.

[29] Kauffold J, Amer HA, Berqfeld U, Weber W, Sobirai A (2005). The *in vitro* developmental competence of oocytes from juvenile calves is related to follicular diameter. J Reprod Dev.

[30] Motlik J, Fulka J (1976). Breakdown of the germinal vesicle in pig oocytes *in vivo* and *in vitro*. J Exp Zool.

[31] Nagai S, Yasumizu T, Kasai T, Hirata S, Mizuno K, Kato J (1997). Effect of oocyte retrieval from a small leading follicle in fixed-schedule *in vitro* fertilization program. J Obstet Gynaecol Res.

[32] Guthrie HD, Garrett WM (2000). Changes in porcine oocyte germinal vesicle development as follicles approach preovulatory maturity. Theriogenology.

